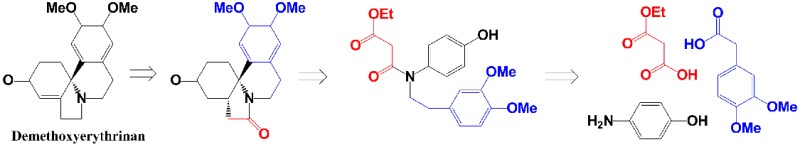# Oxidative dearomatic approach towards the synthesis of erythrina skeleton: a formal synthesis of demethoxyerythratidinone

**DOI:** 10.1007/s13659-011-0045-1

**Published:** 2011-12-23

**Authors:** Zhi-Qiang Pan, Ji-Xuan Liang, Jing-Bo Chen, Xiao-Dong Yang, Hong-Bin Zhang

**Affiliations:** Key Laboratory of Medicinal Chemistry for Natural Resource (Yunnan University), Ministry of Education, School of Chemical Science and Technology, Yunnan University, Kunming, 650091 Yunnan, China

**Keywords:** erythrina alkaloid, oxidative dearomation, demethoxyerythratidinone, formal synthesis

## Abstract

A concise synthetic route leading to highly functional erythrina alkaloid skeletons has been developed. The key process is an oxidative carbon-carbon coupling followed by a conjugated addition. Based on this new strategy, a formal synthesis of demethoxyerythratidinone was completed in only 6 steps from 4-aminophenol.

## Introduction

The genus *Erythrina* is widely distributed in tropical and subtropical regions and has been used in indigenous medicine.[1] Erythrina alkaloids are common constitutes isolated from this species. Representative structures of the erythrina alkaloid family are characterized by a tetracyclic ring system and featured with an 1-azaspiro[5.5]undecane unit (Figure 1). A variety of biological effects are associated with the erythrina skeleton including sedative, hypotensive, neuromuscular blocking and CNS activity.[1a,2] Because of its typical structure and interesting bioactivities, there has been much interest in the synthesis of these alkaloids and derivatives over the years.[3] An impressive strategy towards erythrina skeleton is biomimetic approach through an oxidative carbon-carbon bond formation.[4] Although numerous imaginative synthetic routes have been developed based on the oxidative coupling,[5] few meet adequate measures of flexibility and efficiency in terms of diversity oriented synthesis.

We recently initiated a research program towards the synthesis of structurally diverse compounds by using oxidative formation of carbon-carbon bond as the key reaction. The spiro cyclohexyldienonyl *β*-lactams, oxindoles and structural unit for erythrina alkaloids were synthesized from the amide derivatives of 4-aminophenol.[6] In this paper, we reported the results towards the synthesis of analogues of erythrina alkaloids.

## Results and Discussion

The retrosynthetic analysis is showed in Scheme 1. Starting from the commercially available 4-aminophenol (1kg/157USD),

**Figure 1.** Representative erythrina alkaloids.

we speculated that a highly functional erythrina skeleton could be reached by a sequential oxidative dearomatization and a Michael addition.

We began our research by an amidation of 4-aminophenol (**1**) with homoveratroyl chloride (**2**).[7] The product, amide (**3**), was then converted to amine by a reduction with lithium aluminium hydride in THF (83%). By treatment of amine (**4**) with ethyl 3-chloro-3-oxopropanoate in dichloromethane in the presence of pyridine, amide (**5**) was obtained in 71% yield over three steps. Other amides (**10** and **11**), aiming at the synthesis of erythraline and its analogues, were prepared in three or four steps by the same procedure as shown in Scheme 2.

**Scheme 1.** Retrosynthetic analysis of the erythrina alkaloids.

*To whom correspondence should be addressed. E-mail: zhanghb@ynu.edu.cn; xdyang@ynu.edu.cn

**Scheme 2.** Synthesis of amide intermediates.

The key oxidative coupling process was attempted by treatment of amide (**5**) with iodobenzene diacetate (IBD) in methanol, a well-established dearomatization procedure.[8] To our disappointment, this oxidative condition only led to cyclohexyldienone (**12**). We then tried the oxidation with (bis(trifluoroacetoxy)iodo) benzene (PIFA) in trifluoroethanol at −40°C.[9] To our delight, this oxidation provided the desire azaspiro cyclohexyldienone (**13**), after treatment with DBU in dichloromethane, the desire intermediate (**14**) with a highly functional erythrina skeleton was obtained in 72% yield. This two steps procedure was soon optimized to be an efficient "one-pot" reaction: after the oxidative coupling, potassium carbonate was directly introduced to the reaction mixture at room temperature, and compound **14** was obtained in 83% isolated yield. Although oxidative coupling of amides **10** and **11** proceeded under the same reaction condition, the yield (70% and 72% for **15** and **16**) was relatively lower than that of amide **5**, possibly due to the poor electron donating ability imposed by stereoelectronic effect of the methylenedioxy substituent.[10] This one-pot cyclization furnished the highly functional erythrina skeletons in an efficient way (4 steps for erythrina derivative **14** and 4~5 steps for erythrina derivatives **15** and **16**).

Having successfully constructed the azaspiro cyclohexyldienone, we turned our attention next to convert compound **14** to the natural product, namely 3-demethoxyerythratidinone. Compound **14** was further manipulated, hydrogenation followed by decarboxylation, to a known intermediate for the synthesis of demethoxyerythratidinone (Scheme 4), thus furnished a formal synthesis of this natural erythrina alkaloid.[11]

In conclusion, we have developed a practical and efficient method for the synthesis of erythrina skeletons. Based on this methodology, a formal synthesis of demethoxyerythratidinone was also completed. The highly functional erythrina derivatives could be used not only as intermediates for the synthesis of natural erythrina alkaloids but also be used as building blocks for the synthesis of erythrina alkaloid-like compounds.

**Scheme 3.** Concise synthesis of erythrina skeletons.

**Scheme 4.** Formal synthesis of erythrina alkaloid demethoxyerythratidinone.

## Experimental Section

**General Experimental Procedures**. Melting points were obtained on a XT-4 melting-point apparatus and were uncorrected. Proton nuclear magnetic resonance ($^1$H NMR) spectra were recorded on a Bruker Avance 300 spectrometer at 300 MHz. Carbon-13 nuclear magnetic resonance ($^{13}$C NMR) was recorded on Bruker Avance 300 spectrometer at 75 MHz. Chemical shifts are reported as $\delta$ values in parts per million (ppm) relative to tetramethylsilane (TMS) for all recorded NMR spectra. Low-resolution mass spectra were recorded on a VG Auto Spec-3000 magnetic sector MS spectrometer. High resolution mass spectra were taken on AB QSTAR Pulsar mass spectrometer. Silica gel (200–300 mesh) for column chromatography and silica $GF_{254}$ for TLC were produced by Qingdao Marine Chemical Company (China). All air- or moisture-sensitive reactions were conducted under an argon atmosphere. Starting materials and reagents used in reactions were obtained commercially from Acros, Aldrich, Fluka and were used without purification, unless otherwise indicated.

**Synthesis of Compound 3.** To a solution of 4-aminophenol (**1**, 546 mg, 5 mmol) in $CH_2Cl_2$ (20 mL) and pyridine (2.02 mL, 25 mmol, 5.0 eq.) at 0°C, homoveratroyl chloride (**2**, 5.5 mmol, 1.1 eq.) in dichloromethane (5 mL) was added dropwise. The resulting mixture was then stirred at room temperature for 3–4 h. The reaction mixture was diluted with $CH_2Cl_2$ (20 mL) and treated with 2N HCl (10 mL) for 10 min and the aqueous phase was separated and the organic phase was washed with water (2 × 15 mL). After dried over anhydrous $Na_2SO_4$ and filtration, the solvent was removed under reduced pressure and the residue (1.43 g) was used in next step without further purification.

**Synthesis of Compound 4.** To a solution of **3** (1.43 g) in anhydrous THF (50 mL) at 0°C, $LiAlH_4$ (380 mg, 10 mmol, 2.0 eq.) was added in one portion. The resulting mixture was gradually warmed up to room temperature then stirred at 75°C overnight. The reaction mixture was cooled to 0°C and an aqueous solution of saturated $K_2CO_3$ (5 mL) was added dropwise. The mixture was diluted with water (50 mL) and extracted with EtOAc (3 × 15 mL). The organic phases were combined and washed with water (2 × 15 mL) and brine (15 mL). After dried over anhydrous $Na_2SO_4$ and filtration, the solvent was removed under reduced pressure and the residue was chromatographed on silica gel ($CH_2Cl_2$ : EtOAc = 15:1) to afford amine **4** (984 mg, 72% over two steps). Pale yellow syrup, $^1$H NMR (300 MHz, $CD_3COCD_3$) $\delta$: 6.82 (1H, d, $J$ = 8.1 Hz), 6.80–6.63 (4H, m), 6.54 (2H, d, $J$ = 8.4 Hz), 3.85 (3H, s), 3.84 (3H, s), 3.32 (1H, t, $J$ = 6.6 Hz), 2.83 (1H, t, $J$ = 6.6 Hz). $^{13}$C NMR (75 MHz, $CD_3COCD_3$) $\delta$: 148.98, 148.71, 147.60, 141.46, 131.86, 120.78, 116.36, 115.43, 112.16, 111.52, 55.96, 55.87, 46.61, 34.93. HRESIMS $m/z$ found: 274.1437, calcd. for $C_{16}H_{20}NO_3$ $[M + H]^+$: 274.1443.

**Synthesis of Compound 5.** To a solution of **4** (820 mg, 3 mmol) in $CH_2Cl_2$ (30 mL) and pyridine (1.21 mL, 15 mmol, 5.0 eq.) at 0°C, ethyl 3-chloro-3-oxopropanoate (496 mg, 3.3 mmol, 1.1 eq.) in $CH_2Cl_2$ (3 mL) was added dropwise. The resulting mixture was then stirred at room temperature for 2–3 h. The reaction mixture was then treated with 2N HCl (10 mL) for 10 min and diluted with $CH_2Cl_2$ (20 mL). The aqueous phase was separated and the organic phase was washed with water (2 × 3 mL). The organic phases were combined and back extracted with EtOAc (10 mL). The combined organic phases were dried over anhydrous $Na_2SO_4$ and filtration. After removal of the solvent under reduced pressure, the residue was chromatographed on silica gel (Hexane : EtOAc = 4:1–2:1) to afford amides **5** (815 mg, 70%). Pale yellow powder, m.p.: 138–139ºC. $^1$H NMR (300 MHz, $CDCl_3$) $\delta$: 6.93 (2H, d, $J$ = 8.7 Hz), 6.90 (2H, d, $J$ = 8.7 Hz), 6.75 (1H, d, $J$ = 7.8 Hz), 6.74 (1H, d, $J$ = 1.5 Hz), 6.68 (1H, dd, $J$ = 7.8, 1.5 Hz), 4.10 (2H, q, $J$ = 7.2 Hz), 3.89 (2H, t, $J$ = 7.9 Hz), 3.81 (6H, s), 3.20 (2H, s), 2.83 (2H, t, $J$ = 7.9 Hz), 1.20 (3H, t, $J$ = 7.2 Hz). $^{13}$C NMR (75 MHz, $CDCl_3$) $\delta$: 167.97, 167.14, 157.11, 148.97, 147.64, 133.75, 131.09, 129.17, 120.87, 116.73, 112.22, 111.42, 61.51, 55.98, 55.92, 51.50, 41.90, 33.37, 14.09. HRESIMS $m/z$ found: 388.1759, calcd. for $C_{21}H_{26}NO_6$ $[M + H]^+$: 388.1760.

**Synthesis of Compounds 8 and 9.** The procedures were same as compounds **3** and **4**. Compound **8** (52%): Pale yellow syrup. $^1$H NMR (300 MHz, $CD_3COCD_3$) $\delta$: 6.59 (1H, d, $J$ = 8.1 Hz), 6.55–6.51 (4H, m), 6.34 (2H, d, $J$ = 8.4 Hz), 5.76 (2H, s), 3.12 (1H, t, $J$ = 6.9 Hz), 2.64 (1H, t, $J$ = 6.9 Hz), 0.83 (9H, s), 0.00 (9H, s). $^{13}$C NMR (75 MHz, $CD_3COCD_3$) $\delta$: 147.82, 1447.54, 146.12, 142.54, 133.19, 121.66, 120.71, 114.20, 109.12, 108.32, 100.87, 46.11, 35.37, 25.79, 18.20, −4.50. HRESIMS $m/z$ found: 371.1925, calcd. for $C_{21}H_{29}NO_3Si$ $[M]^+$: 371.1917. Compound **9** (33%): Pale yellow syrup, $^1$H NMR (300 MHz, $CD_3COCD_3$) $\delta$: 6.75 (1H, d, $J$ = 7.8 Hz), 6.70–6.63 (4H, m), 6.53 (2H, d, $J$ = 8.7 Hz), 5.93 (2H, s), 3.29 (1H, t, $J$ = 6.9 Hz), 2.80 (1H, t, $J$ = 6.9 Hz). $^{13}$C NMR (75 MHz, $CD_3COCD_3$) $\delta$: 147.97, 148.80, 146.11, 142.05, 133.08, 121.65, 116.25, 114.77, 109.09, 108.32, 100.86, 46.35, 35.25. HRESIMS $m/z$ found: 257.1057, calcd. for $C_{15}H_{15}NO_3$ $[M]^+$: 257.1052.

**Synthesis of Compound 10.** To a solution of **8** (557 mg, 1.5 mmol) in $CH_2Cl_2$ (20 mL) and pyridine (0.6 mL, 7.5 mmol, 5.0 eq.) at 0°C, ethyl 3-chloro-3-oxopropanoate (248 mg, 1.65 mmol, 1.1 eq.) in $CH_2Cl_2$ (3 mL) was added dropwise. The resulting mixture was then stirred at room temperature for 2–3 h. The reaction mixture was then treated with 2N HCl (10 mL) for 10 min and diluted with $CH_2Cl_2$ (20 mL). The aqueous phase was separated and the organic phase was washed with water (2 × 3 mL). The organic phases were combined and back extracted with EtOAc (10 mL). The combined organic phases were dried over anhydrous $Na_2SO_4$ and filtration. Then removal of the solvent under reduced pressure and dissolved in dry THF (20 mL). To the solution was added TBFA (1.0 M solution in THF, 1.5 mL, 1.5 mmol) under argon. The reaction mixture was stirred at room temperature for 2 h. The mixture was concentrated and diluted with water (30 mL) and extracted with EtOAc (3 × 15 mL). The organic phases were combined and washed with water (2 × 15 mL) and brine (15 mL). After dried over anhydrous $Na_2SO_4$ and filtration, the solvent was removed under reduced pressure and the residue was chromatographed on silica gel (Hexane : EtOAc = 4:1–2:1) to afford amine **10** (512 mg, 92% over two steps). Pale yellow powder. $^1$H NMR (300 MHz, $CDCl_3$) $\delta$: 8.10 (1H, s), 7.00 (2H, d, *J* = 8.7 Hz), 6.94 (2H, d, *J* = 8.7 Hz), 6.68 (1H, d, *J* = 7.8 Hz), 6.65 (1H, d, *J* = 1.2 Hz), 6.60 (1H, dd, *J* = 1.2, 7.8 Hz), 5.88 (1H, s), 4.12 (2H, q, *J* = 8.1 Hz), 3.86 (2H, t, *J* = 8.4 Hz), 3.23 (2H, s), 2.81 (2H, t, *J* = 8.1 Hz), 1.25 (3H, t, *J* = 7.2 Hz). $^{13}$C NMR (75 MHz, $CDCl_3$) $\delta$: 167.91, 167.19, 157.11, 147.64, 146.09, 133.58, 132.12, 129.01, 121.68, 116.75, 109.22, 108.22, 100.81, 61.47, 51.66, 41.79, 33.45, 14.01. HRESIMS *m*/*z* found: 371.1369, calcd. for $C_{20}H_{21}NO_6$ $[M]^+$: 371.1369.

**Synthesis of Compound 11.** To a solution of **9** (386 mg, 1.5 mmol) in toluene (20 mL) 2,2,6-trimethyl-1,3-dioxin-4-one (213 mg, 1.5 mmol) in toluene (5 mL) was added dropwise. The resulting mixture was then stirred at reflux overnight. The mixture was concentrated and the residue was chromatographed on silica gel (Hexane : EtOAc = 4:1–2:1) to afford amine **11** (423 mg, 90%). Pale yellow powder. $^1$H NMR (300 MHz, $CDCl_3$) $\delta$: 7.91 (1H, s), 6.95 (2H, d, *J* = 8.7 Hz), 6.91 (2H, d, *J* = 8.7 Hz), 6.72 (1H, d, *J* = 8.1 Hz), 6.66 (1H, d, *J* = 1.2 Hz), 6.61 (1H, dd, *J* = 1.2, 8.1 Hz), 5.91 (1H, s), 3.87 (2H, q, *J* = 7.8 Hz), 3.33 (2H, s), 2.81 (2H, t, *J* = 7.8 Hz), 2.06 (3H, s). $^{13}$C NMR (75 MHz, $CDCl_3$) $\delta$: 202.64, 167.77, 156.90, 147.65, 146.09, 133.82, 132.13, 129.09, 121.70, 116.70, 109.23, 108.23, 100.82, 51.39, 50.13, 33.52, 30.33. HRESIMS *m*/*z* found: 341.1259, calcd. for $C_{19}H_{19}NO_5$ $[M]^+$: 341.1263.

**Synthesis of Compound 14.** To a solution of **5** (387 mg, 1.0 mmol) in $CF_3CH_2OH$ (3 mL) was added PIFA (473 mg, 1.1 mmol, 1.1 eq.) at −40°C under $N_2$. After stirring at −40°C for 2 h, the reaction mixture was allowed to warm to room temperature then a powder of $K_2CO_3$ (456 mg, 3.3 mmol, 3.3 eq.) was added. The resulting mixture was filtered and washed with EtOAc (10 mL). The filtrate was concentrated and the crude products were chromatographed on silica gel ($CH_2Cl_2$ : EtOAc = 10:1) to provide enone **14** (318 mg, 83%). White crystals, m.p.: 161–162ºC. $^1$H NMR (300 MHz, $CDCl_3$) $\delta$: 6.71 (1H, dd, *J* = 10.5, 1.8 Hz), 6.67 (1H, s), 6.65 (1H, s), 6.18 (1H, d, *J* = 10.2 Hz), 4.41 (1H, dd, *J* = 12.0, 3.9 Hz), 4.30–4.21 (2H, m), 3.87 (3H, s), 3.79 (3H, s), 3.39 (1H, d, *J* = 11.7 Hz), 3.21 (1H, dd, *J* = 12.1, 5.7 Hz), 3.20–2.93 (3H, m), 2.72 (1H, d, *J* = 12.9 Hz), 2.66 (1H, d, *J* = 12.9 Hz ), 1.27 (3H, t, *J* = 7.2 Hz). $^{13}$C NMR (75 MHz, $CDCl_3$) $\delta$: 195.07, 168.29, 166.65, 149.23, 148.49, 145.79, 127.65, 126.75, 126.30, 112.79, 108.77, 62.19, 59.49, 56.36, 56.09, 54.03, 44.45, 36.69, 35.73, 29.03, 14.29. HRESIMS *m*/*z* found: 386.1599, calcd. for $C_{21}H_{24}NO_6$ $[M + H]^+$: 386.1603.

**Synthesis of Compounds 15 and 16.** The procedures were same as compound **14**. Compound **15** (70%): Pale yellow syrup. $^1$H NMR (300 MHz, $CDCl_3$) $\delta$: 6.69 (1H, dd, *J* = 10.2, 1.8 Hz), 6.67 (1H, s), 6.66 (1H, s), 6.17 (1H, d, *J* = 10.2 Hz), 5.96 (2H, s), 4.40 (1H, dd, *J* = 12.0, 3.9 Hz), 4.31–4.20 (2H, m), 3.38 (1H, d, *J* = 11.7 Hz), 3.21 (1H, m), 3.13–2.89 (3H, m), 2.70 (1H, d, *J* = 12.9 Hz), 2.65 (1H, d, *J* = 12.9 Hz ), 1.31 (3H, t, *J* = 7.2 Hz). $^{13}$C NMR (75 MHz, $CDCl_3$) $\delta$: 194.82, 168.17, 166.51, 147.64, 147.17, 145.38, 127.81, 126.64, 127.31, 109.85, 105.57, 101.47, 62.07, 59.63, 53.89, 44.39, 36.34, 35.54, 29.46, 14.15. HRESIMS *m*/*z* found: 369.1220, calcd. for $C_{20}H_{19}NO_6$ $[M]^+$: 369.1220. Compound **16** (72%): Pale yellow syrup. $^1$H NMR (300 MHz, $CDCl_3$) $\delta$: 6.71 (1H, s), 6.68 (1H, dd, *J* = 10.2, 1.8 Hz), 6.45 (1H, s), 6.15 (1H, *d*, *J* = 10.2 Hz), 5.95 (2H, s), 4.34 (1H, dd, *J* = 12.0, 3.9 Hz), 3.48 (1H, d, *J* = 11.4 Hz), 3.30–3.24 (1H, m), 3.13–3.01 (2H, m), 2.88 (1H, ddd, *J* =16.8, 12.3, 5.1 Hz), 2.69 (1H, dd, *J* = 15.9, 2.1 Hz), 2.54 (1H, d, *J* = 18.3 Hz ), 2.47 (3H, s). $^{13}$C NMR (75 MHz, $CDCl_3$) $\delta$: 201.74, 195.45, 166.55, 147.58, 147.18, 145.65, 127.68, 127.49, 109.74, 105.55, 101.45, 59.87, 59.13, 41.48, 36.72, 35.42, 30.97, 29.37. HRESIMS *m*/*z* found: 339.1104, calcd. for $C_{19}H_{17}NO_5$ $[M]^+$: 339.1104.

**Synthesis of Compound 17.** To a solution of **13** (385.4 mg, 1.0 mmol) in anhydrous EtOH (20 mL) was added a powder of 10% Pd/C (38 mg). The resulting mixture was then stirred at room temperature under a balloon of hydrogen overnight. After fitration and washed with $CH_2Cl_2$ (5 mL), the filtrate was concentrated and the residue was chromatographed on silica gel ($CH_2Cl_2$ : EtOAc = 5:1–3:1) to afford ketone **17** (368 mg, 95%). White plates, m.p.: 182–183ºC. $^1$H NMR (300 MHz, $CDCl_3$) $\delta$: 6.68 (1H, s), 6.56 (1H, s), 4.34 (1H, dd, *J* = 13.2, 5.4 Hz), 4.15 (2H, q, *J* = 7.2 Hz), 3.86 (3H, s), 3.84 (3H, s), 3.44–3.34 (1H, m), 3.18 (1H, d, *J* = 8.1 Hz), 3.15–2.90 (2H, m), 2.65 (1H, d, *J* = 16.8 Hz), 2.64 (1H, d, *J* = 16.8 Hz), 2.49–2.20 (4H, m), 1.22 (3H, t, *J* = 7.2 Hz). $^{13}$C NMR (75 MHz, $CDCl_3$) $\delta$: 209.23, 169.01, 167.18, 148.71, 148.54, 133.87, 125.48, 111.94, 107.36, 62.16, 61.47, 56.44, 56.03, 55.21, 42.55, 42.06, 35.62, 35.44, 33.50, 27.82, 14.14. HRESIMS *m*/*z* found: 388.1751, calcd. for $C_{21}H_{26}NO_6$ $[M + H]^+$: 388.1760.

**Synthesis of Compound 18.** A mixture of **17** (193 mg, 0.5 mmol.), LiCl (21 mg, 0.5 mmol, 1.0 eq.) and $H_2O$ (27 mg, 1.5 mmol, 3.0 eq.) in DMSO (15 mL) was stirred at 158°C under $N_2$ overnight (ca. 15–17 h). The resulting mixture was then concentrated under reduced pressure and the residue was chromatographed on silica gel ($CH_2Cl_2$ : EtOAc = 3:1–2:1) to afford **18** (118 mg, 75%). Pale yellow powder, m.p.: 164–165ºC. $^1$H NMR (300 MHz, $CDCl_3$) $\delta$: 6.68 (1H, s), 6.56 (1H, s), 4.37 (1H, dd, *J* = 12.0, 5.4 Hz), 3.86 (3H, s), 3.84 (3H, s), 3.15–2.90 (4H, m), 2.78–2.53 (3H, m), 2.49–2.20 (4H, m), 2.11 (1H, dd, *J* = 17.8, 7.5 Hz). $^{13}$C NMR (75 MHz, $CDCl_3$) $\delta$: 210.13, 172.19, 148.60, 148.43, 134.48, 125.65, 111.91, 107.45, 62.56, 56.45, 56.03, 43.38, 37.90, 37.59, 35.35, 34.84, 33.64, 27.67. HRESIMS *m*/*z* found: 316.1556, calcd. for $C_{18}H_{22}NO_4$ $[M + H]^+$: 316.1548.

**Acknowledgments**

This work was supported by grants (20925205, 21062026, 2010GA014 and 2009CB522300) from National Natural Science Foundation of China, Yunnan Province and National Basic Research Program of China (973 Program).

**Open Access** This article is distributed under the terms of the Creative Commons Attribution License which permits any use, distribution, and reproduction in any medium, provided the original author(s) and source are credited.
